# Ancient and modern: hints of a core post‐transcriptional network driving chemotherapy resistance in ovarian cancer

**DOI:** 10.1002/wrna.1432

**Published:** 2017-08-01

**Authors:** Sarah Blagden, Mai Abdel Mouti, James Chettle

**Affiliations:** ^1^ Department of Oncology University of Oxford Oxford UK

## Abstract

RNA‐binding proteins (RBPs) and noncoding (nc)RNAs (such as microRNAs, long ncRNAs, and others) cooperate within a post‐transcriptional network to regulate the expression of genes required for many aspects of cancer behavior including its sensitivity to chemotherapy. Here, using an RBP‐centric approach, we explore the current knowledge surrounding contributers to post‐transcriptional gene regulation (PTGR) in ovarian cancer and identify commonalities that hint at the existence of an evolutionarily conserved core PTGR network. This network regulates survival and chemotherapy resistance in the contemporary context of the cancer cell. There is emerging evidence that cancers become dependent on PTGR factors for their survival. Further understanding of this network may identify innovative therapeutic targets as well as yield crucial insights into the hard‐wiring of many malignancies, including ovarian cancer. *WIREs RNA* 2018, 9:e1432. doi: 10.1002/wrna.1432

This article is categorized under:

1 RNA Interactions with Proteins and Other Molecules > Protein–RNA Interactions: Functional Implications
2 Translation > Translation Mechanisms
3 RNA in Disease and Development > RNA in Disease

## INTRODUCTION

Epithelial ovarian cancer (EOC) is diagnosed in approximately 239,000 women every year worldwide. It is the most lethal of gynecological cancers; only 45% of those with the disease remain alive 5 years after their diagnosis.^1^ The most significant contributor to its high mortality is that, owing to its subtle presenting symptoms, EOC is predominantly diagnosed at an advanced stage but also has a high rate of recurrence. Initial treatment comprised of surgery and chemotherapy is usually effective at inducing remission but, in 70–80% patients, the cancer recurs within 2 years. At this point re‐treatment with chemotherapy becomes increasingly futile as the cancer cells become more ‘chemoresistant.’^2^

Unlike many cancers with an average of 30–60 mutations each, EOC has a relatively bland mutational profile. According to The Cancer Genome Atlas (TCGA) Research Network data only nine nonsynonymous gene mutations are present in high‐grade serous ovarian cancer (HGSOC), the most common histological subtype. The two most frequent are *TP53* and *BRCA* mutations, present in 96 and 22%, respectively.^3^ The other mutations are present in only 2–6% cases. Although poly(adenosine diphosphate (ADP)‐ribose) polymerase (PARP) inhibitors have transformed the management of those with hereditary BRCA mutations and p53‐targeted therapies are in clinical trials, the low penetrance of the others limits the utility of mutation‐driven approaches.^4^, ^5^ HGSOC has a high degree of chromosomal instability (CIN), attributed to mutations and promoter methylations in DNA homologous repair genes. High CIN is a predictor of greater sensitivity to DNA‐damaging chemotherapies like platinum agents.^3^, ^6^, ^7^ Tumors like EOC with low mutational burden show limited response to immune checkpoint inhibitors, such as those designed to target PD1 and PDL1.^8^ This is borne out in current clinical practice where studies of checkpoint inhibitors as monotherapies in EOC have shown limited efficacy.^9^ For these reasons, cytotoxic chemotherapy remains the mainstay of treatment for non‐BRCA mutated ovarian cancer patients, in the primary (newly diagnosed) context and at relapse.

## CHEMOTHERAPY RESISTANCE

Two chemotherapies predominate in the clinical management of ovarian cancer: the pseudoalkylating agent carboplatin and the microtubule spindle poison paclitaxel. Carboplatin is derived from cisplatin and functions by inducing cell damaging cross‐links between DNA, RNA, and protein in rapidly dividing cells. Paclitaxel functions by binding β tubulin microtubules and preventing spindle remodeling during cell division, resulting in mitotic arrest.^10^ Resistance mechanisms to paclitaxel include point mutations or alterations in the expression of β‐tubulin isotypes such as upregulation of the β3‐tubulin (TUBB3) isoform and overexpression of the efflux protein P‐glycoprotein 1 (encoded by the gene *MDR1*) that exports paclitaxel from the cell.^11^, ^12^ For carboplatin (and other platinum agents such as cisplatin) whose cellular uptake is reliant on heavy metal pathways, resistance mechanisms include down‐regulation of the copper channel influx transporters CTR1 and CTR2, upregulation of the efflux transporter ATP7B, increased DNA damage repair, and enhanced survival signaling.^13^, ^14^ It is likely that at diagnosis ovarian cancer contains a proportion of innately resistant cells that gain clonal dominance with each recurrence and utilize multiple resistance mechanisms.^15^ Therapies to successfully reverse resistance remain elusive and, for patients with recurrent disease, death from progressive disease is unfortunately inevitable.

There is now an accumulating body of evidence that therapeutic opportunities lie within the realm of post‐transcriptional biology.

## POST‐TRANSCRIPTIONAL GENE REGULATION (PTGR)

The former central dogma of molecular biology was that nucleic acids regulate protein in an irreversibly linear sequence.^16^ However, comparisons between gene and protein expression within cells and more recently within HGSOC itself have revealed that the ratio of mRNA to protein is not 1:1 and the abundance of a particular cellular protein cannot necessarily be predicted from the copy number of its encoding gene.^17^, ^18^ It is now understood that post‐transcriptional factors bind mRNAs and modify (and in some cases dominate) the amount of protein they can generate.^19^ These regulatory factors have been identified as noncoding RNAs (ncRNAs) and RNA‐binding proteins (RBPs). Both influence protein expression by controlling the maturation, modification (including stability and translation efficiency), subcellular transport, and/or degradation of their target mRNAs.^20^ While it was once assumed these two classes of PTGR factors worked in isolation they are now known to act co‐operatively. Pier Paolo Pandolfi was the first to describe a competing endogenous (ce)RNA network, initially around microRNAs but later including other ncRNAs, that collectively coordinates gene expression.^21^ Although current descriptions of the ceRNA network include RBPs, we will refer here to this cooperation between RBPs and ncRNAs as the ‘PTGR network.’ In cancer, it is apparent that a PTGR network is co‐opted to regulate the expression of genes that participate in pathological processes such as cell migration, invasion, and metastasis. Advances in computational biology have allowed the collation of expression profiling datasets in common cancers and their correlation with known, validated interactions to generate putative ceRNA network maps. In EOC these maps have focused around microRNAs, lncRNAs and mRNAs but are yet to include RBPs.^22^, ^23^ Here we discuss RBPs that have been identified and known to be deregulated in ovarian cancer and their interactions with other PTGR factors.

### RNA Binding Proteins

**Figure 1:**
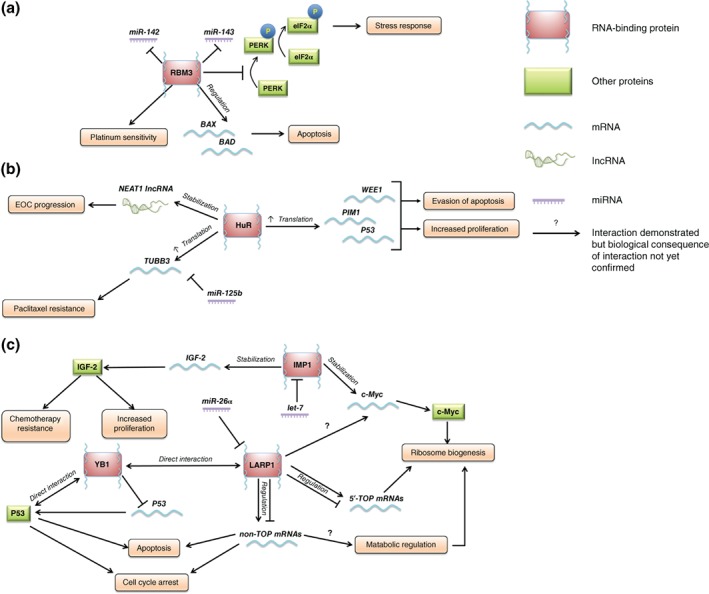
(a)–(c) RNA‐binding proteins influence epithelial ovarian cancer (EOC) progression through complex networks with mRNAs, noncoding RNAs, and other proteins. (a) RNA‐binding motif protein 3 (RBM3) regulates platinum sensitivity and patient survival through regulation of mRNAs involved in apoptosis and the stress response. (b) HuR exerts an oncogenic effect through stabilization and therefore increased translation of a range of mRNAs. (c) RNA‐binding proteins, such as YB1, LARP1, and IMP1 may converge on multiple subsets of mRNAs and signaling pathways as part of a network that drives progression of EOC and/or resistance to chemotherapy.

From the moment mRNA transcripts are synthesized they become bound to RBPs that are involved in every stage of their lifespan.^20^ Although RBPs can be found bound at many sites along an mRNA transcript, they tend to bind within the untranslated regions (UTRs) of mRNA transcripts. Some bind to conserved sequences [such as adenylate‐uridylate (AU)‐rich elements], others recognize structural conformations like stem loops or hairpin bends.^24^, ^25^ RBPs have RNA binding domains, such as RNA recognition motifs (RRM) and hnRNP K‐homology domains.^26^ While the first RBPs to be identified were members of the canonical eIF4F cap‐binding complex, other RBPs have since been characterized, including those with noncanonical roles in specialized protein synthesis.^27^ There are now an estimated 1542 genes encoding RBPs.^28^ This has led to comparisons of the differential levels and patterns of RBP expression between normal and cancer cells. Cellular levels of RBP genes are significantly higher than ncRNAs suggesting RBPs have a more prominent role in PTGR.^29^ Although RBPs function in the synthesis of all proteins, they have an important role in cellular stress by selectively modulating gene expression to provide a stress response. Thus, RBPs are important for disease prevention in normal cells but their deregulation can contribute to many pathological conditions such as inflammation and cancer.^30^ For this reason, and because they bind multiple mRNAs within a single physiological or pathological pathway, RBPs have been identified as potential drug targets.^19^ Recent advances in drug discovery have led to an expanded repertoire of what is considered ‘druggable’ and now includes gene depletion by anti‐sense oligonucleotides and RNA intereference as well as inhibitors of protein–protein, and protein–RNA interactions in addition to classical kinase inhibitors.^31^ This, combined with preclinical evidence of efficacy but minimal toxicity from serendipitous RBP inhibitors like the eIF4E inhibitor ribavirin, has highlighted the feasibility of targeting RBPs.^32^ However, RBPs do not act alone and there is accumulating evidence of interactions with other PTGR network factors to be considered (Figure 1).

### Noncoding RNAs

It is estimated that <2% of the human genome is stably transcribed into protein, the rest is believed to transcribe mRNAs that do not encode proteins and are termed noncoding RNAs (ncRNAs). NcRNAs are classified by size into long noncoding RNAs (lncRNAs) of over 200 and small RNAs with fewer than 200 nucleotides. While some ncRNAs like transfer, ribosomal and spliceosomal RNAs have established cellular housekeeping functions and are constitutively expressed, others including lncRNAs, microRNAs (miRNAs or miRs), small interfering RNAs (siRNAs), and PIWI‐interacting RNAs (piRNAs) were initially perceived as transcriptional ‘junk.’ However, as detailed in a number of reviews, they are now recognized as having important regulatory roles in gene expression.^33^, ^34^, ^35^, ^36^ New classes of small RNAs have more recently been defined such as transfer RNA‐related fragments (tRFs), pseudogenes, and circular RNAs (circRNAs) although these have yet to be fully characterized in EOC.^37^, ^38^, ^39^

#### Long Noncoding RNAs

LncRNAs range from 200 to 100,000 nucleotides in length. They have some overlapping characteristics with mRNA being transcribed by RNA Polymerase II and are (usually) 5′ capped and polyadenylated. However, unlike mRNAs, they do not have a typical open reading frame, are usually shorter, and are expressed at lower levels.^40^ Importantly, lncRNAs are processed and regulated differentially and appear to have expression patterns that are specific to subcellular site, cell type, developmental stage, or disease. Approximately 23,000 lncRNAs have been described so far, of which only a few have been shown to have important roles in cellular regulation and fewer still have been linked to cancer.^41^ LncRNAs act by multiple mechanisms such as altering chromatin remodeling, binding and regulating transcription factors and acting as protein–protein interaction scaffolds. Through these mechanisms, lncRNAs fine‐tune protein expression and in cancers act as oncogenes, tumor suppressors, or as both depending on the circumstances. Intriguingly, some lncRNAs have been shown to act as microRNA sponges and carry microRNA response elements (MREs) to bind and sequester miRNAs away from their intended mRNA targets.^42^ LncRNAs have also been identified as RBP‐sponges, an area of discovery in its infancy as data from Photoactivatable Ribonucleoside‐Enhanced Crosslinking and Immunoprecipitation (PAR‐CLIP), individual‐nucleotide resolution UV crosslinking and immunoprecipitation (iCLIP), and other RBP‐immobilization methods become available.^43^ Those such as *MALAT1*, *HOST2*, *PVT1 NEAT1*, and *HOTAIR* have been identified in EOC and both *PVT1* and *HOTAIR* have been linked to platinum resistance by regulating apoptosis factors and via NF‐κB activation, respectively.^44^, ^45^, ^46^

#### MicroRNAs

MicroRNAs (miRs) are a class of endogenous, small noncoding RNAs of approximately 22 nucleotides in length that bind and repress translation. It is estimated that each microRNA regulates around 100 genes and that, as PTGR factors, microRNAs fine‐tune protein expression.^47^ They are transcribed from intronic, exonic, or intragenic regions of protein encoding ‘host’ genes, in parallel with host gene expression. A ‘cluster’ of multiple microRNAs can be transcribed from a single gene. After undergoing a series of processing steps, mature single‐stranded microRNA is integrated with Argonaute (Ago) into an RNA induced silencing complex (RISC) which then binds its targets by partial base pairing between the microRNA 5′ ‘seed region’ and the 3′ MRE in the target mRNA. Depending on the complementarity between these two sequences, the target mRNA is either degraded by Ago‐mediated cleavage or attenuated. The latter occurs at translation initiation by the RISC hindering assembly of the eIF4F complex or preventing the recruitment of ribosome components.^48^ Since microRNAs were first discovered, the number to have been identified in human cells has now reached 2500 and it is estimated that around 60% all genes are associated with miRNAs.^49^, ^50^, ^51^, ^52^ For this reason, they are involved in almost all biological processes. In cancer, in which microRNAs are generally downregulated, they contribute to many aspects of the disease, from its initial formation to its chemotherapy responsiveness.^53^, ^54^, ^55^ This is partially because microRNA clusters are often located within genomic regions amplified, deleted, hyper‐, or hypomethylated in cancer.^56^ This is particularly relevant in EOC with its high genomic instability.^57^ Of the 34 miRNAs currently known to be deregulated in cancer, 17 have been associated with HGSOC.^58^, ^59^ Those linked to cisplatin resistance include Let‐7 family members *let‐7e* and *7i* and *miR‐214* and *miR‐30a‐5p*, whereas *miR‐663*, *miR‐622*, and *miR‐130b* regulate paclitaxel resistance via the expression of p53 network genes and *MDR1* respectively.^60^, ^61^, ^62^, ^63^ In addition, *miR‐200c* is associated with reversal of resistance via its regulation of *TUBB3*. Although these studies identify putative target mRNAs that could explain the resistance phenotype, it is highly likely that there are hundreds of other genes regulated by each microRNA that contribute incrementally toward chemotherapy resistance.

As summarized by Ciafrè and Galardi,^64^ RBPs work collaboratively with miRNAs, either by attracting the miRNA‐RISC complex to degrade mRNAs or, if the RBP‐binding or ‘USER’ site on the target mRNA is close or overlapping the MRE, by preventing the interaction between a miRNA and its target mRNA resulting in translation derepression. RBPs can compete with microRNAs by binding and modifying the structure of the target mRNA or by sequestering it away from microRNAs to another location within the cell.

## PATHOLOGICAL RBPS AND THEIR PTGR NETWORKS IN OVARIAN CANCER

The relationships that exist between RBPs and microRNAs, lncRNAs and other ncRNAs have yet to be fully characterized but this is becoming achievable using state‐of‐the‐art computational models like SimiRa (http://vsicb‐simira.helmholtz‐muenchen.de), CircInteractome (circinteractome.nia.nih.gov), doRiNA (dorina.mdc‐berlin.de.), and StarBase (starbase.sysu.edu.cn) that integrate and interrogate data from publically available RNA‐Seq and CLIP datasets to generate interaction maps and/or provide functional categorisations.^65^ The RBPs described below have been previously been characterized in the context of EOC and any known interactions with ncRNAs are outlined (Table 1).

**Table 1:** RNA‐Binding Proteins (RBPs) Described in Epithelial Ovarian Cancer and Their Known Interactions with ncRNAs

RBP	Abbreviation	Family	Biological Role	Role in Epithelial Ovarian Cancer (EOC)	RBP–ncRNA Interaction
RNA‐binding motif protein 3	RBM3	Glycine‐rich RNA binding protein (GRP)^66^	Regulates global protein synthesis under normal physiological conditions and in response to cold temperature and low oxygen tension^114^	Elevated mRNA and protein levels are associated with a favorable prognosis and sensitivity to platinum‐based chemotherapy through post‐transcriptional regulation of the apoptosis mediators BCL2, BAX, and genes involved in DNA integrity as well as impairment of DNA damage repair following chemotherapy^67^	RBM3 regulates the expression of temperature sensitive miR‐142‐5p and miR‐143 to attenuate pathological hyperthermia and downregulates miR‐143‐mediated nitric oxide‐induced apoptosis by interfering with p38 MAPK kinase signaling in human SH‐SY5Y neuroblastoma cells^115^
ELAV‐like protein 1	HuR	Embryonic lethal abnormal visual system (ELAV)^116^, ^117^	Promotes the stability of many AU‐rich element (ARE)‐containing mRNAs^71^ as well as translation of various mRNAs (reviewed in Ref 118) Essential for placental branching morphogenesis and embryonic development^119^	HuR positively regulates stability and/or translation of ZEB2 mRNA which plays a key role in ovarian cancer progression, where concomitant high cytoplasmic HuR and nuclear ZEB2 correlates with unfavorable prognosis^120^	In ovarian cancer, cytoplasmic HuR competes with miR‐200c in binding to TUBB3 mRNA, derepressing TUBB3 expression^79^ HuR antagonizes miR‐125b‐mediated translation repression of TP53 through its binding to TP53 mRNA 3′ UTR during DNA damage^80^ lncRNA NEAT1 expression is enhanced by HuR, but suppressed by miR124‐3p in OVCAR3 ovarian cancer cells^83^ HuR binds the lncRNAs HOTAIR and MALATI, enhancing their miRNA sponge activity^84^, ^85^
IGF2 mRNA‐binding protein 1	IGF2BP1/IMP1	IGF2 mRNA‐binding protein (IMP)^121^	Key regulator of neural crest migration, neurite development, and stem cell properties^122^, ^123^, ^124^, ^125^ Stabilizes its target mRNAs during cellular stress, transiently forming stress granules^126^	Stabilizes mRNAs that drive drug resistance such as c‐myc and MDR1^91^, ^92^, ^93^	IMP1 is a downstream target gene of let‐7 miRNA. Let‐7 negatively regulates IMP1 expression,^95^, ^96^ increasing the sensitivity of resistant ovarian cancer to Taxanes^92^
Y‐box binding protein 1	YBX1/YB‐1	Cold‐shock domain containing proteins^99^	Transcription, translation, DNA damage repair,^127^, ^128^ mRNA stability and translational regulation in the cytoplasm^129^, ^130^, ^131^	Nuclear expression positively correlates with poor prognosis^132^ and cisplatin resistance^133^	miR‐190a negatively regulates mRNA and protein levels of YB‐1 in prostate cancer^103^
La‐related protein 1	LARP1	La‐related protein (LARP)^104^	Regulates the stability and/or translation of mRNAs required for ribosome biogenesis and cell survival and proliferation^106^, ^107^	Promotes EOC progression and resistance to chemotherapy through post‐transcriptional regulation of cell survival mRNAs such as BCL2 and BIK^108^, ^109^	LARP1 is directly regulated by miRNA‐26a/b, inhibiting cancer cell invasion in prostate cancer^111^ lncRNA TGFB2‐OT1 derepresses LARP1 expression in vascular endothelial cells through its binding to miR‐4459^113^

### RNA‐Binding Motif Protein 3

RNA‐binding motif protein 3 (RBM3) is a member of the glycine‐rich RNA binding protein (GRP) family. Transcript levels of RBM3 are elevated in cell lines immediately following cold shock (when taken from 37 to 32°C) and RBM3 is often co‐expressed with another GRP family member, cold‐inducible RNA‐binding protein.^66^ Levels of RBM3 have subsequently shown to be elevated across multiple cancers, generally in association with a favorable outcome. In the context of EOC, mRNA and protein levels of RRM3 were correlated with outcome in tissue collected from 163 and 151 patients with EOC respectively. Messenger RNA levels of RBM3 were found to be an independent predictor of better relapse‐free and overall survival while presence (vs absence) of RBM3 protein was strongly associated with prolonged overall survival. Higher RBM3 (mRNA or protein) was associated with increased platinum‐sensitivity, attributed to its interactome of apoptosis regulating mRNAs such as *BCL‐2*, *BAX* as well as genes involves in DNA integrity; upregulation of RBM3 impaired DNA damage repair after chemotherapy and improved its cytotoxic effect.^67^ RBM3 also inhibits PERK phosphorylation and prevents cell death from endoplasmic reticulum (ER) stress.^68^ In addition, RBM regulates the biogenesis of temperature sensitive miRNAs such as *miR‐142* and *miR‐143* but has not yet been associated with any oncomiRs or lncRNAs.^69^

### HuR

The ubiquitously expressed mRNA‐binding protein HuR (ELAVL1) is one of the four membered embryonic lethal abnormal visual (ELAV) system family of proteins expressed in human cells.^70^ In embryos, these evolutionarily conserved proteins are involved in neuronal development. In normal adult cells HuR is expressed at low levels and shuttles from the nucleus into the cytoplasm in response to stress where it stabilizes and/or promotes the translation of mRNAs containing AU‐rich elements within their 3′ UTRs.^71^ In cancers, elevated levels of cytoplasmic HuR *positively* correlate with treatment resistance and adverse survival outcome, attributed to its target mRNAs such as *PIM1*, the DNA damage‐regulated G2 checkpoint gene *Wee1*, the TNF‐related apoptosis‐inducing ligand (TRAIL) component *DR4* and the cell survival regulator *TP53*.^72^, ^73^, ^74^, ^75^, ^76^, ^77^, ^78^ In addition, HuR is linked to paclitaxel resistance as it binds and stabilizes the mRNA encoding TUBB3 in competition with *miR‐200c*. When HuR is nuclear, *miR‐200c* binds the 3′ UTR of cytoplasmic *TUBB3* inhibiting it and conferring a good prognosis, but when HuR is cytoplasmic it competes with *miR‐200c* and the expression of *TUBB3* is derepressed.^79^ Similarly, HuR has been shown to compete with *miR‐125b* in binding the 3′ UTR of *TP53* during DNA damage preventing *miR‐125b* from reaching its MRE site.^80^ Control of the shuttling of HuR between the cytoplasm and the nucleus is a crucial component of its regulation. In a recent study using murine macrophages, HuR was shown to be bound and PARylated by the DNA damage‐repair protein PARP1 following immune stimulation. This post‐translational modification (the addition of a polymeric poly‐ADP ribose chain) of HuR appears to stabilize its interaction with target mRNAs in the cytoplasm.^81^

HuR interacts with lncRNAs such as nuclear enriched abundant transcript 1 (*NEAT1*). *NEAT1* is up‐regulated in EOC where it is associated with more advanced disease and poorer prognosis.^82^ In ovarian cancer cell lines, ectopic expression of *NEAT1* promotes cell proliferation and invasion. Work by Chai et al. in OVCAR3 ovarian cells showed that HuR binds and stabilizes *NEAT1* and increases its expression. However, the microRNA *miR124‐3p* competes with HuR to destabilize *NEAT1*.^83^ In other cancers, HuR has been shown to bind the lncRNAs HOX transcript antisense intergenic RNA (*HOTAIR*) and metastasis‐associated lung adenocarcinoma transcript‐1 (*MALAT1*) enhancing their activity as microRNA sponges.^84^, ^85^ *HOTAIR* is upregulated in patients with EOC where it drives proliferation, migration, and invasion and acts as a sponge for *miR‐373*, thereby derepressing the Ras oncogene family member *Rab22a*.^86^ Levels of *MALAT1* have been shown to be elevated in a number of cancers including EOC and, in SKOV3 cells, *MALAT1* drives proliferation, invasion, and tumorigenicity.^87^ The mechanism of action for *MALAT1* in EOC has remained elusive although it has been shown, in endometrial cancer, to sponge *miR‐200c* thus derepressing *TUBB3* expression.^88^ More recent studies in EOC lines have demonstrated *MALAT1* acts as a sponge to *miR‐506* and derepresses the apoptosis inhibitor *iASPP*.^89^ It is therefore likely that, in EOC, HuR drives oncogenic behavior and chemoresistance through direct interactions with its target mRNAs and indirectly via its PTGR network.

### IGF2 mRNA‐Binding Protein 1 (IGF2BP1/IMP1)

The IMP family is comprised of IMP 1–3 and is so named because its members bind to the 5′‐UTR of insulin‐like growth factor 2 (IGF2) mRNA. They are important for embryogenesis and (apart from IMP2) are expressed at low levels in adult cells but all three paralogues are highly expressed in cancers where levels are associated with poor prognosis.^90^ IMP1 modulates the turnover of target mRNAs during stress by trafficking mRNAs to cytosolic mRNP complexes, presumably to protect them from degradation. In addition to its eponymous target *IGF2*, IMP1 stabilizes *c‐myc* as well as the Adenosine triphosphate (ATP)‐dependent efflux pump *MDR1* and thus has been implicated in ovarian chemoresistance.^91^, ^92^, ^93^ RNAi knockdown of *IMP‐1* in cell lines from several types of cancers reduces c‐Myc levels, inhibits cell proliferation, and triggers apoptosis.^94^ IMP1 is negatively regulated by the microRNA *let7* and cooperates within an ‘oncogenic triangle’ between IMP1 and two other *let‐7* targets (the RBP) LIN28B and the transcriptional regulator HMGA2.^95^, ^96^ Although an inhibitory interaction between IMP1 and *lncMyoD* has been postulated in skeletal muscle cells, there are currently no documented interactions between IMP1 and lncRNAs in cancer.^97^

### Y‐Box Binding Protein 1 (YBX1/YB1)

YB‐1 is a member of an evolutionarily conserved family of cold‐shock domain containing proteins.^98^, ^99^ YB‐1 is essential for normal embryogenesis and levels of the protein are high in embryos but decline in subsequent stages of development.^100^ Initially classified as a transcription factor, YB‐1 was also shown to have RNA‐binding capability by virtue of two RNA‐binding motifs contained within its cold shock domain. It has both nuclear and cytosolic localization and elevated expression of YB‐1 has been observed in many cancers, including EOC where higher levels are correlated with adverse survival outcome. *YB‐1* null cells have enhanced sensitivity to multiple stresses including from genotoxic drugs such as cisplatin. The mechanism behind the YB‐1‐regulated stress response is not yet fully understood and may have transcriptional and post‐transcriptional components. The protein undergoes nuclear accumulation under stress. YB‐1 binds CCAAT elements (Y‐box) in the promoter region of *MD1* and nuclear levels of YB‐1 correlate with expression of P glycoprotein.^101^ Reduction of YB‐1 in colorectal cancer cell lines causes induction of p53‐dependent cell death and *TP53* levels rise after YB‐1 repression. YB‐1 has also been shown to directly bind p53 protein at the site of DNA damage.^102^ As yet YB‐1 has not been associated with ncRNAs in EOC. In prostate cancer, *miR‐190a* has been shown to directly bind and repress *YB‐1*. In advanced prostate cancer *miR‐190a* expression is reduced resulting in *YB‐1* derepression and activation of androgen‐receptor cell signaling.^103^

### La‐Related Protein 1

La‐related protein 1 belongs to a seven‐member family of RBPs called the LARPs. It is highly evolutionarily conserved with orthologues present in all metazoan species.^104^ In *Drosophila*, LARP1 is required for normal embryonic development as well as spermatocyte formation.^105^ In normal human cells LARP1 is predominantly cytoplasmic and expressed at low levels where it acts downstream of mTORC1 to bind and regulate the translation of mRNA transcripts required for ribosome manufacture (biogenesis) and cell proliferation.^106^, ^107^ These are TOP mRNAs characterized by a consensus 5′ terminal oligopyrimidine (TOP) sequence. Levels of LARP1 protein are elevated in a number of epithelial malignancies including EOC and confers adverse survival outcome. LARP1 is required for stress response; cells depleted for LARP1 have heightened sensitivity to genotoxic agents like cisplatin and paclitaxel, as well as other stresses such as hypoxia and glucose starvation. In cancer cell lines, LARP1 has been shown to bind an interactome of approximately 3000 mRNAs that is enriched for transcripts encoding cell survival and RNA biogenesis proteins. *In vitro* and *in vivo* inhibition of LARP1 induces cell death in part through its post‐transcriptional regulation of the apoptosis mediators *BCL2* and *BIK*.^108^, ^109^ Although no consensus binding sequences within its targets have yet been identified, LARP1 binds these mRNAs via its C‐terminal ‘DM15 repeat region’ that adopts a HEAT domain‐like configuration.^110^ Although it has not been associated with ncRNAs in EOC, in breast and prostate cancer LARP1 has recently been shown to be one of several targets of *miR‐26a*.^111^, ^112^ In endothelial cells, the lncRNA *TGFB2‐OT1* acts as a sponge to *miR‐4459* resulting in derepressed expression of LARP1.^113^

## SUMMARY AND FUTURE PERSPECTIVES

It is evident that, as with other cancers, a PTGR network exists in EOC that regulates the malignant characteristics of the disease. There are notable similarities between the RBPs described here. They are all oncofetal genes required for embryonic development that are mobilized during stress in normal adult cells but are highly expressed in cancer where they drive cell migration, invasion, and tumorigenesis. They are also evolutionarily ancient. *RBM3* and *YB1* are ancestral cold‐shock genes, *LARP1*, *HuR*, and *IMP1* belong to gene families that predate eukaryotes. Unlike ncRNAs that have tumor site, type and stage specificity, the RBPs described here have activities that are consistent across multiple cancer types. The majority are proto‐oncogenic and their inhibition induces apoptotic cell death. This indicates not only that these RBPs are influential ‘nodes’ within the PTGR network but also that they have a crucial function in maintaining cancer cell survival.

There is much overlap in the target mRNAs described here. They encode proteins controlling ATPase efflux pumps, copper uptake proteins, tubulin isoforms, and apoptotic regulators implying they belong to a core set of mRNAs that are themselves evolutionarily conserved. As these proteins also control the uptake and efflux of chemotherapy drugs, they are correspondingly and coincidentally associated with treatment resistance. The possibility of the existence of a core set of PTGR targets indicates that analysis of the interactomes of more cancer RBPs (once they are known) may reveal more overlapping candidates. While fully annotating such a complex network may seem daunting, the commonalities between those factors known so far suggests they exist within a single coordinated stress response. This implies that a similar post‐transcriptional response is deployed whatever form of stress the cell is exposed to. If this were proven to be the case, there would be huge therapeutic advantage in targeting a PTGR node when genomic heterogeneity so limits the success of conventional targeted therapies.

Like transcription factors, the nonenzymatic nature of RBPs have earned them the reputation of being ‘undruggable’ and knockdown approaches using RNAi or antisense have been preferred. An example is therapeutic RNAi against HuR which causes enhanced apoptosis and chemosensitivity in EOC cell lines and xenograft models but has yet to reach the clinic.^134^ As it is likely that each RBP is deployed in many different contexts in the normal and cancer cell, a preferable approach may prove to be the blockade of the site of interaction between an RBP and its core target gene(s) or ncRNAs. Fortunately, improvements in drug design have heralded the development of molecules capable of disrupting protein:protein as well as protein:RNA interactions.^135^ To embark on such a drug discovery program for RBPs described here will require a clear understanding of the direct interactome of each RBP, the site of these interactions and their impact on the wider PTGR network. Currently, although the RBPs described here have identifiable RRM, it cannot be assumed that these are the site of interactions with the core mRNAs regulating survival and chemosensitivity. For example, the DM15 region of LARP1, now known to bind cell survival transcripts (such as BCL2 and BIK) is C‐terminally placed and somewhat remote from the N‐terminal La‐RRM (La motif) which is the *de facto* site of mRNA binding in other LARP family members.^104^

As with other cancer types, a comprehensive annotation of the PTGR network in EOC is in its infancy. Although many cancer RBPs have been identified from high throughput screens, few have undergone functional analysis and their target mRNA interactomes are undocumented. This prevents their incorporation into PTGR algorithms and thus limits our understanding of the roles played by RBPs in post‐transcriptional gene networks. As our knowledge of these networks unfolds, it is likely that opportunities for developing new inhibitors will be revealed. Also, the discovery that some RBPs are subject to PARylation and other post‐translational modifications may provide insights into their contribution to the clinical responses that are currently observed with targeted cancer therapies. Here a biased, RBP‐centric review of the published literature surrounding PTGR in ovarian cancer gives fascinating insights into an ancient network that dominates multiple aspects of cancer behavior including chemotherapy resistance.
